# Exercise is associated with younger methylome and transcriptome profiles in human skeletal muscle

**DOI:** 10.1111/acel.13859

**Published:** 2023-05-02

**Authors:** Sarah Voisin, Kirsten Seale, Macsue Jacques, Shanie Landen, Nicholas R. Harvey, Larisa M. Haupt, Lyn R. Griffiths, Kevin J. Ashton, Vernon G. Coffey, Jamie‐Lee M. Thompson, Thomas M. Doering, Malene E. Lindholm, Colum Walsh, Gareth Davison, Rachelle Irwin, Catherine McBride, Ola Hansson, Olof Asplund, Aino E. Heikkinen, Päivi Piirilä, Kirsi H. Pietiläinen, Miina Ollikainen, Sara Blocquiaux, Martine Thomis, Dawn K. Coletta, Adam P. Sharples, Nir Eynon

**Affiliations:** ^1^ Institute for Health and Sport (iHeS) Victoria University Footscray Victoria Australia; ^2^ Novo Nordisk Foundation Center for Basic Metabolic Research, Faculty of Health and Medical Sciences University of Copenhagen Copenhagen Denmark; ^3^ Faculty of Health Sciences and Medicine Bond University Gold Coast Queensland Australia; ^4^ Genomics Research Centre, Centre for Genomics and Personalised Health, School of Biomedical Sciences Queensland University of Technology Brisbane Queensland Australia; ^5^ ARC Training Centre for Cell and Tissue Engineering Technologies Queensland University of Technology (QUT) Brisbane Queensland Australia; ^6^ Max Planck Queensland Centre for the Materials Sciences of Extracellular Matrices Brisbane Queensland Australia; ^7^ School of Health, Medical and Applied Sciences Central Queensland University Rockhampton Queensland Australia; ^8^ Department of Medicine, School of Medicine Stanford University Stanford California USA; ^9^ Genomic Medicine Research Group, School of Biomedical Sciences Ulster University Coleraine UK; ^10^ Sport and Exercise Sciences Research Institute Ulster University Belfast UK; ^11^ Department of Clinical Sciences, Genomics, Diabetes and Endocrinology Unit, Lund University Diabetes Center Lund University Lund Sweden; ^12^ Institute for Molecular Medicine Finland (FIMM) Helsinki University Helsinki Finland; ^13^ Unit of Clinical Physiology Helsinki University Hospital and University of Helsinki Helsinki Finland; ^14^ Obesity Research Unit, Research Program for Clinical and Molecular Metabolism, Faculty of Medicine University of Helsinki Helsinki Finland; ^15^ HealthyWeightHub, Endocrinology, Abdominal Center Helsinki University Hospital and University of Helsinki Helsinki Finland; ^16^ Minerva Foundation Institute for Medical Research Helsinki Finland; ^17^ Department of Movement Sciences, Physical Activity, Sports and Health Research Group KU Leuven Leuven Belgium; ^18^ Department of Medicine, Division of Endocrinology University of Arizona Tucson Arizona USA; ^19^ UA Center for Disparities in Diabetes Obesity and Metabolism University of Arizona Tucson Arizona USA; ^20^ Department of Physiology University of Arizona Tucson Arizona USA; ^21^ Institute of Physical Performance Norwegian School of Sport Sciences Oslo Norway; ^22^ Australian Regenerative Medicine Institute Monash University Clayton Victoria Australia

**Keywords:** aging, cardiorespiratory fitness, DNA methylation, exercise training, human skeletal muscle, meta‐analysis, mRNA expression

## Abstract

Exercise training prevents age‐related decline in muscle function. Targeting epigenetic aging is a promising actionable mechanism and late‐life exercise mitigates epigenetic aging in rodent muscle. Whether exercise training can decelerate, or reverse epigenetic aging in humans is unknown. Here, we performed a powerful meta‐analysis of the methylome and transcriptome of an unprecedented number of human skeletal muscle samples (*n* = 3176). We show that: (1) individuals with higher baseline aerobic fitness have younger epigenetic and transcriptomic profiles, (2) exercise training leads to significant shifts of epigenetic and transcriptomic patterns toward a younger profile, and (3) muscle disuse “ages” the transcriptome. Higher fitness levels were associated with attenuated differential methylation and transcription during aging. Furthermore, both epigenetic and transcriptomic profiles shifted toward a younger state after exercise training interventions, while the transcriptome shifted toward an older state after forced muscle disuse. We demonstrate that exercise training targets many of the age‐related transcripts and DNA methylation loci to maintain younger methylome and transcriptome profiles, specifically in genes related to muscle structure, metabolism, and mitochondrial function. Our comprehensive analysis will inform future studies aiming to identify the best combination of therapeutics and exercise regimes to optimize longevity.

## Graphical abstract

Higher fitness levels were associated with attenuated differential methylation and transcription during aging in human muscle. Furthermore, exercise training targets many of the age‐related transcripts and DNA methylation loci to maintain younger methylome and transcriptome profiles, specifically in genes related to muscle structure, metabolism, and mitochondrial function. Our comprehensive analysis will inform future studies aiming to identify the best exercise regimes to optimize longevity.

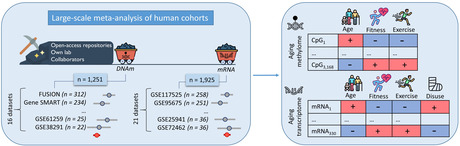

**Abbreviations**
CRF: cardiorespiratory fitness
dbGAP: database of genotypes and phenotypes
DEG: differentially expressed gene
DMG: differentially methylated gene
DMP: differentially methylated position
DNAm: DNA methylation
EWAS: epigenome‐wide association study
FDR: false‐discovery rate
GEO: gene expression omnibus
PC: principal component
TWAS: transcriptome‐wide association study
VO_2max_
: maximum oxygen uptake

## 1 INTRODUCTION

The United Nations has declared 2021–2030 the “Decade of Healthy Ageing” to assist the aging population in living healthier for longer. Identifying reliable aging biomarkers that can be targeted by longevity‐promoting interventions is a global priority, however, requires sizable human cohorts across a broad range of ages and relevant tissues, which is both costly and time‐consuming. The last two decades have seen an open science revolution with the creation of free‐access repositories overflowing with molecular data from all levels of gene regulation (e.g., epigenomics, transcriptomics). These rich repositories allow for the exploration of aging mechanisms and their susceptibility to environmental stressors, even in healthy and/or young individuals, since age‐related changes gradually accumulate from early life and affect organ systems years before disease manifestation (Belsky et al., 2015).

Aging is associated with a loss of muscle mass and function that leads to increased adverse outcomes including falling injury, functional decline, frailty, earlier morbidity, and mortality (Cruz‐Jentoft & Sayer, 2019). Exercise training is one of the most affordable and effective ways to promote healthy aging (Cartee et al., 2016), as being physically active reduces mortality from all causes, independent of levels and changes in several established risk factors (overall diet quality, body mass index, medical history, blood pressure, triglycerides, and cholesterol; Mok et al., 2019). Cardiorespiratory fitness, as estimated by maximal oxygen uptake (VO_2max_) during an exercise test, shows a strong, graded, and inverse association with overall mortality (Laukkanen et al., 2001). However, we have an incomplete understanding of the fundamental mechanisms by which physical activity delays the age‐related decline in skeletal muscle function.

At the molecular level, aging arises from a tip in the balance between cellular damage and compensatory mechanisms (López‐Otín et al., 2023; Thuault, 2021). Cells undergo constant damage, such as genomic instability, telomere attrition, epigenetic alteration, loss of proteostasis, and disabled macroautophagy (López‐Otín et al., 2023). This leads to impaired nutrient sensing, mitochondrial dysfunction, and cellular senescence, which play more nuanced roles in the aging process, as they can be beneficial at a young age (e.g., the nutrient‐sensing network contributes to organ development until young adulthood but can have a detrimental role beyond this stage). Low doses such as occurs in mitochondrial dysfunction can stimulate beneficial counterreactions via mitohormesis, or if spatially confined (e.g., cellular senescence suppression of oncogenesis and improved wound healing; López‐Otín et al., 2023). Eventually, the accumulated damage inflicted by these primary and antagonistic hallmarks can no longer be compensated, leading to stem cell exhaustion, altered intercellular communication, chronic inflammation, and dysbiosis, which are ultimately responsible for the physiological decline associated with aging (López‐Otín et al., 2023). The effect of aging on DNA methylation (DNAm) patterns is so profound that machine learning has spawned highly accurate predictors of both chronological and biological age (termed “epigenetic clocks”; Bell et al., 2019). We developed an epigenetic clock for human skeletal muscle (Voisin et al., 2020) and reported widespread changes in the muscle methylome at genes involved in muscle structure and function (Voisin et al., 2021). Downstream of epigenetic processes, changes in transcriptional patterns at genes involved in central metabolic pathways, and mitochondrial function have also been reported in muscle during aging (Su et al., 2015). Regular exercise mitigates the age‐related loss of proteostasis (Fernando et al., 2019; Ubaida‐Mohien et al., 2019), mitochondrial dysfunction (Cartee et al., 2016; Short et al., 2005), and stem cell exhaustion (Cartee et al., 2016; Sousa‐Victor et al., 2015) in muscle, but there is currently limited evidence for its effect on age‐related epigenetic and transcriptomic changes.

Several cross‐sectional analyses found only weak associations between self‐reported physical activity levels and epigenetic age in blood (Quach et al., 2017; Sillanpää et al., 2019, 2021) or skeletal muscle (Sillanpää et al., 2019), after adjusting for confounders such as diet. However, self‐reported measures of physical activity poorly reflect actual physical activity levels, particularly in individuals with higher body fat and females who typically overestimate energy expenditure (Prince et al., 2008). Furthermore, these studies relied solely on DNAm clocks to quantify age‐related changes in epigenetic patterns. While aging is associated with widespread changes at a plethora of CpG sites, epigenetic age, as measured by epigenetic clocks, is a single value that encompasses a very small portion of the aging methylome (typically a few hundred age‐related DNAm loci, also called CpGs). Therefore, it offers a very narrow and incomplete view of the aging methylome, and exercise training may affect aging regions that are not captured by epigenetic clocks. Interestingly, a recent study found evidence that late‐life exercise mitigates age‐related epigenetic changes in mouse gastrocnemius muscle (Murach et al., 2022). In this study, the authors did not use clocks but investigated all DNAm loci that change with age in mouse muscle and applied a direct exercise training intervention. A couple of human studies are in line with these results: resistance training was shown to offset age‐related changes both in the nuclear (Blocquiaux et al., 2022; Gorski et al., 2023) and mitochondrial (Ruple et al., 2021) epigenome. Unfortunately, these studies had small sample sizes and have not been replicated to date, so they should be regarded as preliminary (Fanelli et al., 2017). They were also restricted to resistance training in males, which does not speak for the effect of exercise training in general in males or females across the lifespan. In another, large‐scale meta‐analysis, age‐related changes in the transcriptome showed an inverse correlation with higher cardiorespiratory fitness (CRF; Su et al., 2015). However, these associations were cross‐sectional and could be confounded by other environmental and lifestyle factors that co‐occur with higher CRF levels (e.g., a better diet). Therefore, there is a great need for a comprehensive, integrative, and robust assessment of the effects of CRF, exercise training, and inactivity on age‐related molecular changes in human muscle.

To address these limitations, we identified relevant published datasets from online databases that we combined with our own original data to characterize the effect of cardiorespiratory (CRF), exercise training, and inactivity on human skeletal muscle aging across the methylome and transcriptome. We first compiled a list of age‐related changes in 1251 samples across 16 cohorts (DNAm) and 1925 samples across 21 cohorts (mRNA expression). We then tested cross‐sectional associations between aging molecular profiles and CRF; we hypothesized that individuals with higher CRF would display “younger” profiles than expected at age‐related CpGs and mRNAs. Then, we investigated directly whether exercise training could shift molecular profiles in human skeletal muscle toward a younger profile, using high‐resolution longitudinal data collected from exercise training studies of various types and durations. Finally, we tested whether inactivity could “age” transcriptomic profiles in human skeletal muscle, using longitudinal data from forced immobilization interventions in humans. This work provides a comprehensive and integrative map of the effect of physical activity on age‐related changes in fundamental processes controlling gene expression in human muscle.

## 2 RESULTS

### 2.1 Methodology overview

**FIGURE 1:**
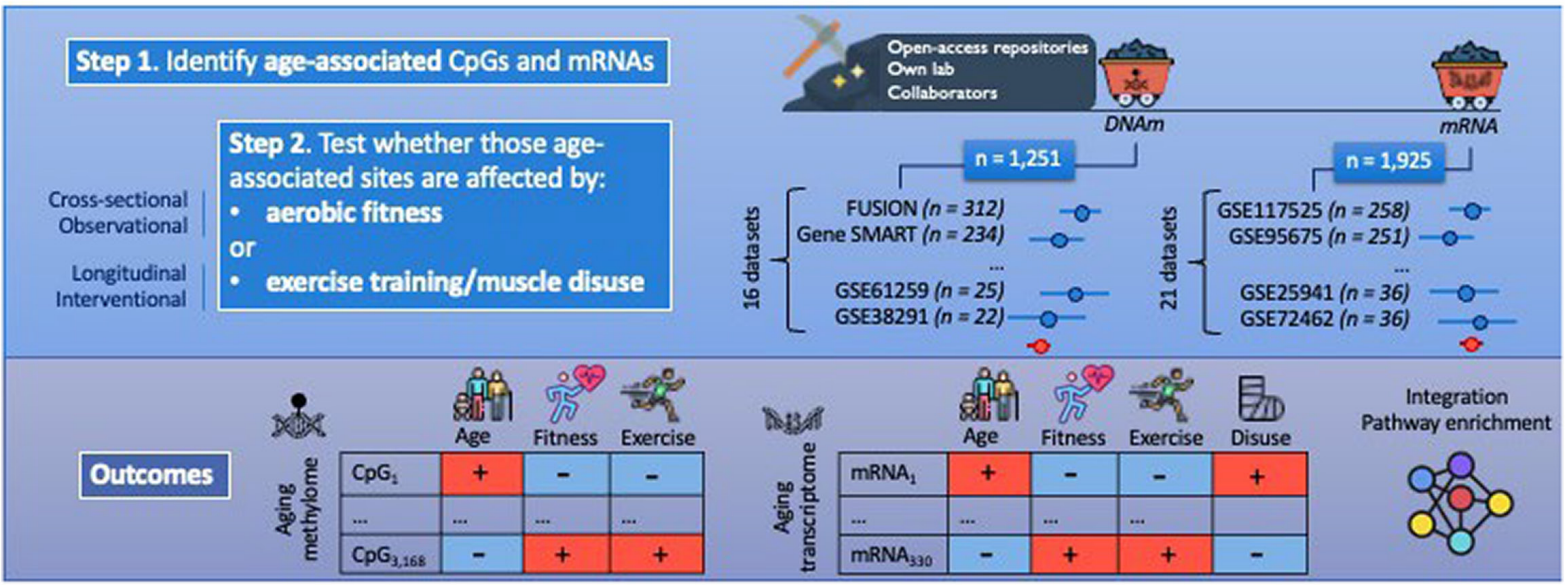
Study overview. First, we performed a large‐scale data mining exercise to gather all existing DNA methylation (DNAm) and mRNA expression microarray datasets from our own lab, our network of collaborators, and open‐access repositories (GEO, dbGAP, ArrayExpress). See Methods for inclusion criteria of datasets. Step 1: We identified age‐related changes in DNAm and mRNA expression in human skeletal muscle by meta‐analyzing 1251 samples across 16 cohorts (DNAm) and 1925 samples across 21 cohorts (mRNA). Step 2: First, we performed a cross‐sectional association between DNAm levels at age‐related CpGs or expression levels at age‐related mRNAs and VO_2max_. Then, we determined whether DNAm levels at age‐related CpGs, and expression levels at age‐related mRNAs changed after exercise training, and we assessed whether expression levels at age‐related mRNAs changed after muscle disuse. Finally, we performed a series of OMIC integrations and pathway analyses to identify the molecular pathways affected by age, VO_2max_, exercise training, and/or muscle disuse across both OMIC layers. Note: we only had OMIC data available at the transcriptomic level following muscle disuse. Note 2: summary statistics for the exercise‐ and disuse‐induced mRNA changes came from two recent meta‐analyses (Fanelli et al., 2017; Garcia et al., 2022).

We describe our study design in Figure 1.

First, we identified the DNAm loci and transcripts that change with age in human skeletal muscle, by systematically mining and analyzing DNAm and mRNA microarray data from our laboratory, online databases, and our collaborators' laboratories. We conducted a random‐effects epigenome‐wide association study (EWAS) meta‐analysis of age across 1251 samples from 16 independent cohorts (Table S1), and a random‐effects transcriptome‐wide association study (TWAS) meta‐analysis of age across 1925 human muscle samples from 21 independent cohorts (Table S2). To uncover the effect of aerobic fitness and exercise training on the aging methylome and transcriptome of skeletal muscle, we then restricted our analysis to the identified age‐related Differentially Methylated Positions (DMPs) and Differentially Expressed Genes (DEGs).

We first assessed whether an objective gold‐standard measure of aerobic fitness (VO_2max_) was associated with younger methylome and transcriptome profiles in skeletal muscle (Figure 1). At the epigenetic level, we conducted a random‐effects meta‐analysis of VO_2max_ across 439 samples from five independent cohorts; at the transcriptomic level, we conducted a random‐effects meta‐analysis of VO_2max_ across 354 samples from five independent cohorts (Table 1). There was a reasonably large range of VO_2max_ in each of these cohorts (SD >5 mL/min/kg, Table 1), which is essential to detect differences in OMIC aging between individuals with varying fitness levels.

**TABLE 1:** Characteristics of the cohorts used in the cross‐sectional analysis (association between age‐related CpGs or mRNAs and maximal oxygen uptake (VO_2max_).

Cohort	Number of samples	Number of unique individuals	Age (years)	Sex (% male)	VO_2max_ (mL/min/kg)	Data availability
*DNA methylation*						
Gene SMART (Voisin et al., 2020, 2021)	234	66	32 ± 8.1	80%	48 ± 9.0	GSE151407, GSE171140
Finnish Twin Cohort (Sillanpää et al., 2021)	73	73	43 ± 16	38%	36 ± 8.5	Per request
E‐MTAB‐11282 (Garcia et al., 2022)	52	13	45 ± 11	38%	23 ± 5.0	E‐MTAB‐11282
EXACT	48	16	33 ± 10	100%	42 ± 7.8	Per request
CAUSE (Gorski et al., 2023)	32	16	60 ± 5.3	0%	30 ± 6.1	GSE213029
*mRNA expression*						
GSE18732 (Gallagher et al., 2010)	117	117	54 ± 11	69%	28 ± 9.6	GSE18732
HERITAGE (Clarke et al., 2017; Takeshita et al., 2021)	82	42	34 ± 14	58%	36 ± 9.0	GSE117070
GSE44818 (Rowlands et al., 2015)	72	12	30 ± 7.2	100%	60 ± 6.0	GSE44818
The Malmö Prevention Study (De et al., 2010)	43	43	66 ± 1.5	100%	28 ± 6.4	E‐CBIL‐30
MSAT (Oskolkov et al., 2022)	39	39	36 ± 8.3	100%	52 ± 8.1	Per request

As cross‐sectional analyses can be confounded by unmeasured factors (e.g., lifelong dietary patterns, socioeconomic status), we tested directly whether an exercise training program could shift OMIC profiles toward a younger state, in an interventional, longitudinal setting. We conducted a random‐effects meta‐analysis of DNAm changes following aerobic, high‐intensity interval, or resistance training across 401 samples from six independent exercise training interventions (Figure 1 and Table 2); we also extracted summary statistics at age‐related DEGs from a published meta‐analysis of transcriptomic changes induced by exercise training (Amar et al., 2021). Finally, and to further support the causal effect of exercise training in the shift of muscle OMIC patterns toward younger profiles, we tested whether age‐related transcriptomic profiles were altered following a *decrease* in physical activity; we extracted summary statistics at age‐related DEGs from a published meta‐analysis following forced immobilization protocols (Pillon et al., 2020).

**TABLE 2:** Participant characteristics from the cohorts in the interventional analysis (changes in levels of age‐related CpGs or mRNA after exercise training or muscle disuse).

Cohort	Number of samples	Number of unique individuals	Age (years)	Sex (% male)	Intervention	Dataset access
*DNA methylation*						
Gene SMART (Voisin et al., 2020, 2021)	196	66	32 ± 8.1	80%	4 weeks of HIIT repeated twice after a > 1‐year washout, and an additional 8 weeks of HIIT	GSE151407, GSE171140
E‐MTAB‐11282 (Garcia et al., 2022)	52	13	45 ± 11	38%	8 weeks of aerobic training	E‐MTAB‐11282
EPIK (Blocquiaux et al., 2022)	48	14	45 ± 22	100%	12 weeks of resistance training repeated twice after a 12‐week washout (older individuals) or 2‐week immobilization (younger individuals)	Per request
GSE114763 (Seaborne et al., 2018)	39	8	29 ± 6	100%	7 weeks of resistance training repeated twice after a 7‐week washout	GSE114763
EpiTrain (Lindholm et al., 2014)	34	17	27 ± 4	44%	3 months of aerobic training	GSE60655
CAUSE (Gorski et al., 2023)	32	16	60 ± 5.3	0%	5 months of aerobic training	GSE213029
*mRNA expression*						
ExTraMeta (Amar et al., 2021)	952	476	Meta‐analysis of 28 datasets		Exercise training of various types and duration	https://www.extrameta.org
MetaMEx (Pillon et al., 2020)	250	125	Meta‐analysis of 7 datasets		Forced immobilization of various durations	https://metamex.serve.scilifelab.se/

### 2.2 Skeletal muscle aging alters DNA methylation and expression of genes involved in muscle structure and metabolism

Age‐related changes to the methylome are widespread yet small (typically ~1% change in methylation per decade of age); out of the 595,541 tested CpG sites, we identified 3168 differentially methylated positions (DMPs) associated with age at FDR <0.005 (Benjamin et al., 2017; Table S3), 73% of which were hypomethylated (Figure S1A). This is in concordance with our previous findings (Voisin et al., 2021), and in line with recent findings at the single‐cell level (Tarkhov et al., 2022). While DMPs were not enriched in any particular canonical pathway or expression signatures of genetic and chemical perturbations, they were overrepresented in two gene ontology terms related to muscle structure (contractile fiber, I band; Figure S1B), and in two human phenotype ontologies entirely consistent with musculoskeletal aging (“difficulty climbing stairs,” and “muscle weakness;” Figure S1B).

Out of the 16,657 tested transcripts, we identified 330 differentially expressed genes (DEGs) at FDR <0.005 (Table S4), 68% of which were downregulated with older age (Figure S2A). While DEGs were not enriched in any particular canonical pathway, they were overrepresented in several gene ontology terms related to mitochondrial function and energy production (ADP and ATP metabolic processes, generation of precursor metabolites and energy, energy derivation by oxidation of organic compounds, mitochondrial protein‐containing complex, organelle inner membrane; Figure S2B). Furthermore, they were also overrepresented in expression signatures of genetic and chemical perturbations (incl. genes up‐regulated in differentiating myoblasts upon expression of *PPARGC1A*; Mootha et al., 2003), genes differentially regulated in myoblasts with *IGF2BP2* knockdown (Boudoukha et al., 2010), and genes that comprise the mitochondria gene module (Wong et al., 2008). DEGs were also enriched in two human phenotype ontologies related to muscle function (“exercise intolerance”, and “myoglobinuria”; Figure S1B).

Finally, we estimated whether the age‐related DNAm and mRNA changes were possibly driven by changes in cell type composition. We tested whether the DMPs and DEGs were overrepresented in twelve gene sets containing curated cluster markers for cell types identified in a single‐cell sequencing study of human skeletal muscle (Rubenstein et al., 2020). We found no significant enrichment of DMPs in any of the cell type marker genes (Figure S1C), suggesting that the age‐related DNAm changes are not confounded by changes in cell type proportions. In contrast, DEGs were enriched for genes whose expression differs between type I and type IIa fibers (Figure S2C), with a change in mRNA expression suggestive of an increase in type I fiber % with older age; *TPM3*, *PDLIM1*, and *MYOZ2* are all markers of type I fibers and increased in expression with age, while *PKM*, *ENO3*, *and PFKM* are all markers of type IIa fibers and decreased in expression with age. DEGs also showed a trend for enrichment in marker genes of smooth muscle cells that make up the walls of blood vessels (Figure S2C), but it is unclear whether it reflected an increase or a decrease in the proportion of smooth muscle cells, as the signal was inconsistent: 17/28 of the marker genes increased in expression, and 11/28 decreased in expression.

As the DMPs and DEGs were associated with related yet distinct ontologies, we further examined the overlap between differentially methylated genes (DMGs) and DEGs. We identified 63 genes that were altered both at the epigenetic and transcriptional levels during aging (Figure S3A and Table S5). This overlap between age‐related changes at the epigenetic and transcriptomic levels was greater than expected by chance alone, as DMGs were more likely to also be DEGs than non‐DMGs (*p*‐value for over‐representation = 0.00011, Figure S3B); conversely, DEGs were more likely to be DMGs than non‐DEGs (*p*‐value for over‐representation $=1.7\times 10^{-5}$, Figure S3B).

### 2.3 CRF and exercise training are associated with younger epigenetic and transcriptomic profiles in human skeletal muscle, in contrast to muscle disuse

We identified 25 age‐related DMPs and one age‐related DEGs that showed a significant association with VO_2max_ (FDR <0.005, Tables S3 and S4), but it is likely that we were underpowered to detect more associations at a high level of confidence. A quantile‐quantile plot of *p*‐values for the association between VO_2max_ and DNAm or mRNA levels at age‐related DMPs or DEGs showed a clear increase (inflation) above the diagonal line (Figure S4A,B). This diagonal line indicates the expected *p*‐value distribution under the assumption (null hypothesis) that the *p*‐values follow a uniform [0,1] distribution (i.e. that there are no true associations between VO_2max_ and DNAm or mRNA levels at age‐related DMPs and DEGs). The amount of departure from this diagonal line correlates with the expected number of true associations (van Iterson et al., 2017). We did not identify age‐related DMPs that were significantly altered following exercise training at FDR <0.005, but with substantially more samples (*n* = 952 from 28 datasets) and therefore statistical power at the transcriptional level, we found 40 age‐related DEGs that were altered following exercise training (Table S3). Importantly, at age‐related DMPs and DEGs most significantly associated with VO_2max_ or altered following exercise training (the points on the far right of the Q–Q plots), VO_2max_ and training were associated with changes that directly countered the effect of age (Figure S4C,D). In other words, DMPs or DEGs whose methylation or expression levels decreased with age overwhelmingly increased in methylation or expression with higher VO_2max_ and following exercise training; DMPs or DEGs whose methylation or expression levels increased with age overwhelmingly decreased in methylation or expression with higher VO_2max_ and following exercise training. Finally, we observed a very pronounced effect of muscle disuse on the aging transcriptome, despite a substantially lower sample size (*n* = 250 from 7 datasets, Table 2): 68 age‐related DEGs were significantly altered following forced immobilization at FDR <0.005 (Table S4 and Figure S4E). DEGs that were downregulated with age tended to decrease in expression following muscle disuse, and vice versa.

**FIGURE 2:**
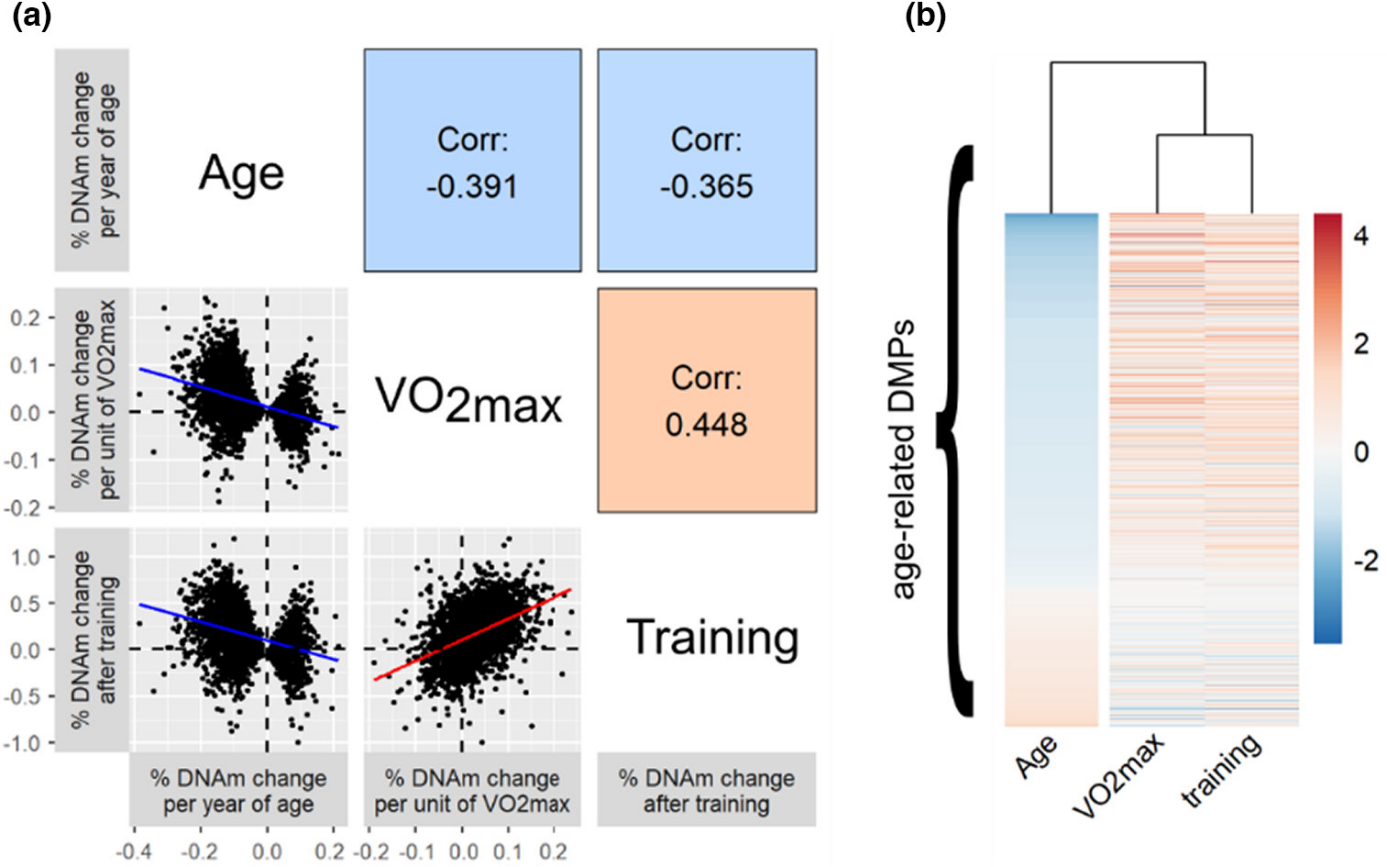
Aerobic fitness and exercise training have similar associations with muscle epigenetic profiles, which contrast with those seen with age. (a) Pairwise correlation (Spearman) between the effect sizes of age, aerobic fitness (VO_2max_), and exercise training at age‐related DMPs. Each dot corresponds to one of the 3168 age‐related DMPs, and the axes represent the magnitude of effect for age, VO_2max_, and exercise training. (b) Unsupervised hierarchical clustering of the effect sizes of age, VO_2max_, and exercise training at age‐related DMPs (ordered from the most hypomethylated to most hypermethylated with age). Note that the legend is arbitrary as effect sizes were scaled to an SD of 1 for age, VO_2max_, and exercise training to be comparable.

**FIGURE 3:**
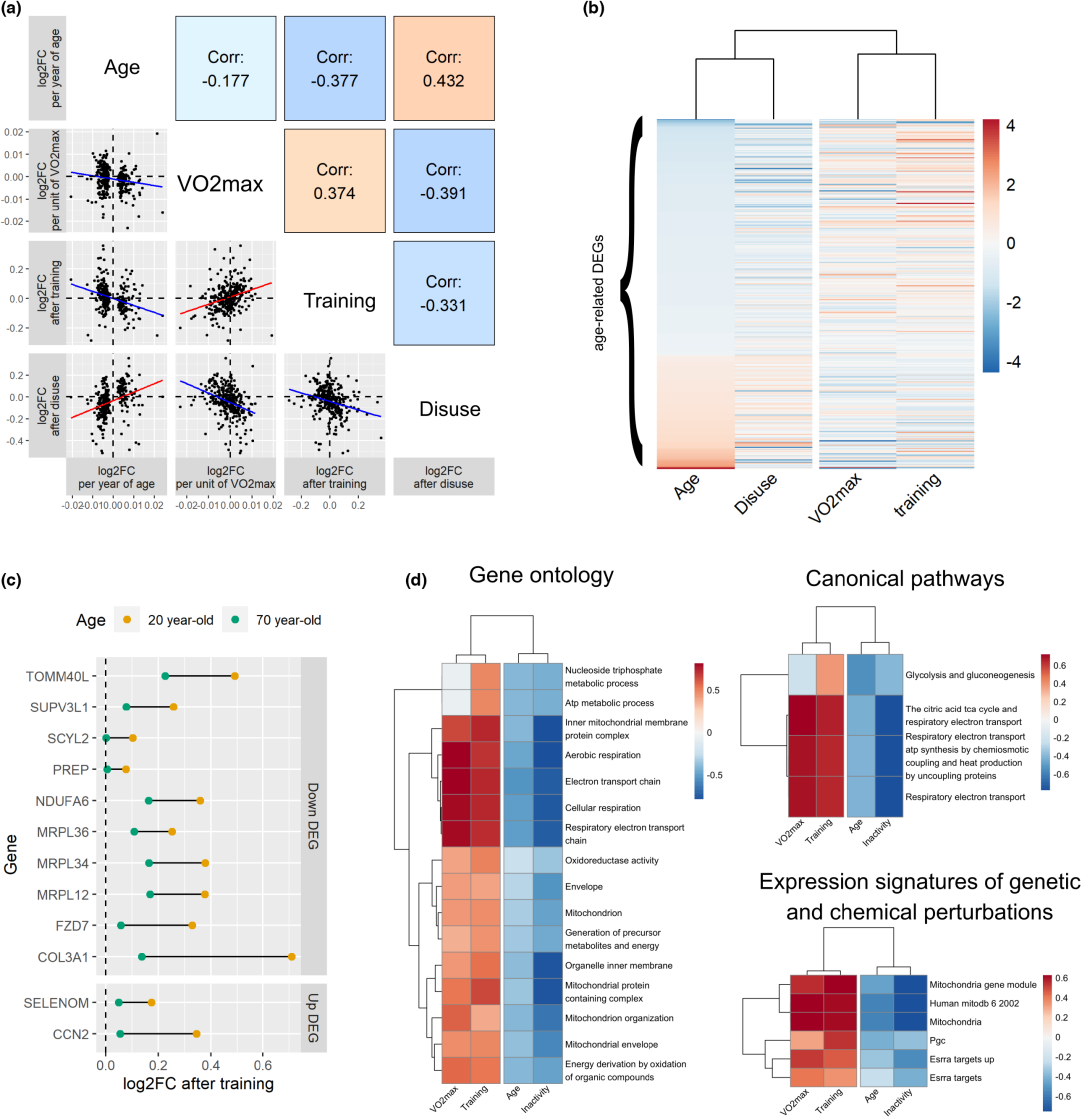
Aerobic fitness and exercise training show similar associations with muscle transcriptomic profiles and contrast with those seen with age and muscle disuse. (a) Pairwise correlation (Spearman) between the effect sizes of age, aerobic fitness (VO_2max_), exercise training, and muscle disuse at age‐related DEGs. Each dot corresponds to one of the 330 age‐related DEGs, and the axes represent the magnitude of effect for age, VO_2max_, exercise training, and disuse. (b) Unsupervised hierarchical clustering of the effect sizes of age, VO_2max_, exercise training, and disuse at age‐related DEGs (ordered from the most downregulated to most upregulated with age). Note that the legend is arbitrary as effect sizes were scaled to an SD of 1 for age, VO_2max_, exercise training, and disuse to be comparable. (c) Comparison of the magnitude of exercise‐induced changes in gene expression between a hypothetical 20‐ and 70‐year‐old. The genes displayed are those for which “age” was a significant moderator according to the meta‐regression conducted by Amar et al. (2021) “Down DEG” = gene whose expression decreases during normal aging; “Up DEG” = gene whose expression increases during normal aging. (d) Multi‐contrast enrichment comparing the effects of age, VO_2max_, exercise training, and muscle disuse at age‐related DEGs. Genes related to mitochondrial function showed clear indications of downregulation during aging and following muscle disuse while being simultaneously upregulated with higher VO_2max_ and following exercise training. This was visible across the Gene Ontology, Canonical Pathways, and Expression Signature of Genetic and Chemical Perturbations gene sets.

The contrast between age‐related and VO_2max_‐ and exercise‐related changes are visible when all age‐related DMPs and DEGs were taken into account; we noted strong negative correlations between the effects of age and VO_2max_ across all age‐related DMPs (Figure 2a, Spearman correlation ρ = −0.39, $p<2.2\times 10^{-16}$, Figure 2b), and across all age‐related DEGs (Figure 3a, Spearman correlation ρ = −0.18, p=0.0013, Figure 3b), as well as strong negative correlations between the effects of age and exercise training overall age‐related DMPs (Figure 2a, Spearman correlation ρ = −0.37, $p<2.2\times 10^{-16}$) and across all age‐related DEGs (Figure 3a, Spearman correlation ρ = −0.38, $p=6.7\times 10^{-12}$). The “pro‐aging” effect of muscle disuse was visible at the scale of the whole aging transcriptome; we found a strong positive correlation between the effects of age and immobilization overall age‐related DEGs (Figure 3a, Spearman correlation ρ = 0.43, $p<2.2\times 10^{-16}$).

**FIGURE 4:**
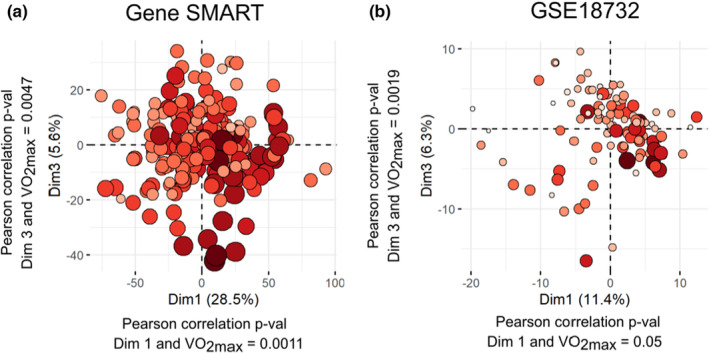
Principal component analysis of individuals from the Gene SMART cohort at age‐related DMPs, and individuals from the GSE18732 cohort at age‐related DEGs. We used principal component analysis (PCA) to reduce dimensionality and show each individual on a two‐dimensional graph. Individuals from the Gene SMART cohort (a) and from the GSE18732 cohort (b) are colored according to their baseline VO_2max_ levels. Lower levels of VO_2max_ are indicated by smaller circles in lighter colors, while higher levels are indicated by larger circles in darker reds. To objectively test the clustering of individuals according to VO_2max_, we ran Pearson correlations between individual coordinates on Dimension 1, Dimension 2, or Dimension 3 and VO_2max_.

To further highlight the association between VO_2max_ and muscle OMIC aging, we performed principal component analysis (PCA) for DMPs in the Gene SMART cohort, and for DEGs in the GSE18732 cohort. These two cohorts have the largest sample size and VO_2max_ range in our study (Table 2). In the Gene SMART cohort, individuals clustered by VO_2max_ levels both on Dimension 1 and Dimension 3 (Pearson correlation p=0.0011 for PC1 and p=0.0047 for PC3), indicating that individuals of similar fitness levels show similar patterns of DNAm at age‐related DMPs (Figure 4a). Similarly, in the GSE18732 cohort, individuals tended to cluster by VO_2max_ levels both on Dimension 1 and Dimension 3 (Pearson correlation p=0.05 for PC1 and p=0.0019 for PC3), indicating that individuals of similar fitness levels have similar mRNA levels at age‐related DEGs (Figure 4b).

### 2.4 Older individuals have a blunted response to exercise at a small fraction of age‐related DEGs

We then explored whether the effects described above were applicable to the entire lifespan (i.e. whether older individuals were as able as young individuals to reap the “anti‐aging” benefits of exercise training). We could not test this hypothesis at the DNAm level due to the more limited number of older individuals with DNAm data in the studied cohorts (Tables 1 and 2), but we investigated this in the transcriptional response to exercise training as effect sizes came from 28 different cohorts with a wide variability in age range (Table 2). We extracted summary statistics from the original paper from Amar et al. (2021) and identified 12 DEGs (3% of all DEGs) whose exercise‐induced change in expression levels depends on age. For all 12 DEGs, older individuals showed a blunted response to exercise training (Figure 3c).

### 2.5 Mitochondrial and metabolic pathways are simultaneously inhibited by age and muscle disuse, and enhanced with greater CRF and by exercise training

We compared the associations between age, aerobic fitness, exercise training, and muscle disuse in an integrated, multi‐contrast enrichment analysis that uses a rank‐MANOVA‐based statistical approach (Kaspi & Ziemann, 2020). We could only perform this multi‐contrast analysis at the transcriptional level, as this statistical approach has not been adapted for DNAm data (i.e., it has not been optimized to take into account the severe bias in gene‐set analysis applied to genome‐wide methylation data; Maksimovic et al., 2021; Phipson et al., 2016). This multi‐contrast analysis identified mitochondrial and metabolic pathways as simultaneously inhibited by age and muscle disuse, while enhanced by aerobic fitness and exercise training (Figure 3d). The effects of VO_2max_ and exercise training were highly consistent both at the epigenetic and transcriptomic levels (Figures 2 and 3). Conversely, the effect of muscle disuse contrasted with both VO_2max_ and exercise training (Figures 2 and 3).

## 3 DISCUSSION

We showed, in a large sample (>3200) of human muscles, that higher aerobic fitness is associated with younger epigenetic and transcriptomic profiles. In line with this, exercise training shifts the aging muscle epigenome and transcriptome toward a younger profile, while muscle disuse significantly accelerates transcriptomic aging. The magnitude of “anti‐aging” or “aging” effects was highly site‐ and gene‐dependent, suggesting that exercise training can reverse specific OMIC changes occurring during normal aging in human skeletal muscle. Transcriptomic integration revealed a pronounced deterioration of mitochondrial function and energy production during aging, which is accelerated by forced immobilization but restored by exercise training.

Although there were few age‐related DMPs and DMGs significantly associated with CRF or exercise training, a close inspection of all statistical tests performed suggested that this was more likely due to a lack of statistical power than an absence of true associations. In support of this, we observed a striking similarity between the effect sizes of aerobic fitness and exercise training on the aging muscle methylome and transcriptome, which directly contrasted with the effects of age and forced immobilization. Adults lose about ~2.5–3 mL/min/kg of VO_2max_ per decade of age (Rapp et al., 2018), partly because of primary aging (i.e. the inevitable deterioration of cellular structure and biological function, independent of disease or harmful lifestyle or environmental factors; Holloszy, 2000), but also due to a decline in physical activity levels as we age (Hallal et al., 2012). Therefore, the anti‐aging effect of exercise and the aging effect of disuse are perhaps unsurprising, given that some of the age‐related signals captured in the EWAS and TWAS meta‐analysis of age reflect a decline in physical activity levels, rather than primary aging per se. This exemplifies the complex nature of aging biology and highlights the challenge of assessing which factors mutually affect each other when causality becomes circular (Cohen et al., 2022; e.g., aging leads to a decline in physical activity/fitness levels, and a decline in physical activity/fitness levels leads to aging; Booth et al., 2011). In a small study of older men, master athletes showed hypomethylation in the promoter of genes involved in energy metabolism and muscle structure, compared with men who reported lifelong sedentary behaviour (Sailani et al., 2019). However, without a young control group, this study could not distinguish the age‐related changes that can be counteracted by exercise, from those that remain unaffected by even the most extreme exercise regimes. Without adjusting for other environmental confounders, it is also unclear whether these effects come from the exercise regime or rather from the effects of other lifestyle factors that correlate strongly with high physical activity levels (e.g., a healthy diet). In our study, the interventional data (i.e. exercise training & muscle disuse protocols) entirely supported the cross‐sectional findings (i.e. associations with CRF), suggesting that the association between CRF and OMIC aging is due to exercise training rather than other unmeasured confounding factors.

We used a large‐scale data mining approach to achieve an unprecedented sample size (>1200 epigenetic profiles and >1900 transcriptomic profiles across 37 cohorts). We applied random‐effects meta‐analyses allowing the effects of age, fitness, exercise, and disuse to vary between cohorts while maintaining the specificity of each dataset (i.e., we did not force a normalization across datasets). Our results may therefore be applicable to a broad range of individuals (sex, health/training status) and exercise regimes (training type/duration). However, the cohorts profiled for DNAm included a majority of healthy, young/middle‐aged, male individuals, so we cannot confidently extrapolate the DNAm results to all populations. It is possible that older, or diseased individuals show blunted responses to exercise training, or that specific training regimes (e.g., endurance vs resistance training) lead to anti‐aging effects that are entirely specific to that training regime. We could only test this at the transcriptional level, and we found that a few age‐related DEGs showed a blunted response to exercise training in older individuals.

While we observed the anti‐aging effect of exercise training at both the epigenetic and transcriptomic levels, and despite a significant overlap between DMPs and DEGs, the molecular pathways affected by age were distinct between the two OMIC layers. It may be due to differences in gene coverage between the DNAm and mRNA arrays, or it could reflect differences in aging mechanisms at the epigenetic and transcriptomic levels. Nevertheless, the integration of all effects at the transcriptomic level clearly showed a downregulation of mitochondrial and energy metabolism pathways during aging and following muscle disuse, which was restored by aerobic fitness and exercise training. This is in line with the known beneficial effect of exercise on mitochondrial function (Goh et al., 2021). The integration was not feasible at the DNAm level because a given CpG can be annotated to multiple genes, and a given gene can harbor multiple CpGs, severely biasing the statistical test for enrichment (Maksimovic et al., 2021; Phipson et al., 2016).

A major challenge in OMIC analysis is determining whether changes are due to a modification of the intrinsic profiles of the cells, or to a change in the relative proportions of different cell types in the sample. We could not directly estimate the proportions of different cell types in our samples, as deconvolution algorithms have currently not been developed for human skeletal muscle, whether at the methylation (Zhu et al., 2022) or transcriptional level. We, therefore, tested for an enrichment of DMGs and DEGs in genes whose expression differs between muscle cell types (Rubenstein et al., 2020). While we found no evidence of confounding by cell type in the epigenetic analysis, some of the age‐related changes in mRNA were indicative of an increase in the proportion of type I fibers (vs type II fibers). This is surprising given that age does not affect the relative proportion of different fiber types, but rather the size and distribution of fibers within the muscle (Deschenes, 2004). Furthermore, we observed younger OMIC patterns in individuals of higher aerobic fitness levels, yet fitter individuals typically harbor greater proportions of type I fibers (Stuart et al., 2013). Therefore, it is likely that the DNAm and mRNA expression changes were intrinsic to muscle cells, rather than reflecting a shift in the proportions of different cell types within the muscle. To answer this question, future studies should investigate the effects of age, aerobic fitness, exercise training, and muscle disuse on OMIC profiles within individual cell types using cell sorting, or single‐cell methods.

We avoided using epigenetic clocks in this analysis for multiple reasons. There are only two clocks currently available that could be applied to muscle DNAm data, namely the Horvath pan‐tissue clock (Horvath, 2013) and the MEAT clock we recently developed for human muscle (Voisin et al., 2020; all other clocks were developed for non‐muscle tissue). First, both the pan‐tissue and MEAT clocks were trained to predict *chronological* age, which is a poor proxy for clinically relevant measures of biological age (this has been highlighted by others; Bell et al., 2019; Field et al., 2018; Zhang et al., 2019) and is the reason for the development of second‐generation clocks, such as PhenoAge (Levine et al., 2018) and GrimAge (Lu et al., 2019, 2022), clocks better adapted to longitudinal data such as DunedinPoAm (Belsky et al., 2020) and DunedinPACE (Belsky et al., 2022), and clocks able to disentangle damaging and adaptive changes during aging (Ying et al., 2022). There are currently too few DNAm datasets with corresponding measurements of muscle function (e.g., mitochondrial function, contractile properties, etc.) to develop an epigenetic clock that would capture muscle biological age. Second, the pan‐tissue clock is poorly calibrated in skeletal muscle, and most of the muscle datasets from the present study were used to generate the MEAT clock (Voisin et al., 2020). This means that epigenetic age estimations using the MEAT clock would be severely biased and unsuitable to assess the effects of fitness and exercise on epigenetic aging. Finally, until recently (Higgins‐Chen et al., 2022), epigenetic clocks only selected a limited number of CpGs to maximize prediction accuracy, which means they would discard information at many potentially relevant CpGs associated with age. We adopted a broader perspective to look at the entire aging methylome and examine the effect of exercise training on this aging trend. We were unable to assess the functional effects of the age‐related OMIC changes on muscle structure, function, and metabolism, so we cannot firmly conclude that the effect of exercise training led to gains in muscle function or quality. Future studies combining OMIC profiles with genetic data to implement Mendelian Randomization analyses (Ying et al., 2022) that would determine whether OMIC aging leads to a decline in muscle function and whether the beneficial effects of fitness and exercise training on muscle function are mediated by a reversal of OMIC aging.

In conclusion, using an unprecedented number of epigenetic and transcriptomic human muscle profiles, meta‐analyses, and OMIC integration, we demonstrated the power of exercise training in shifting the epigenome and transcriptome toward a younger state. We hope that this work will inspire future studies to look deeper at the mechanisms underlying this shift of muscle epigenetic and transcriptomic patterns toward younger profiles.

## 4 METHODS

This study was a large‐scale investigation of the effect of exercise training on the aging muscle methylome and transcriptome in humans. We used a wide range of bioinformatics and computational techniques (data mining, epigenome‐wide association studies, transcriptome‐wide association studies, random effects meta‐analysis, overrepresentation analysis, and multi‐contrast enrichment analysis) to analyze and interpret large amounts of OMIC data in human muscle. By exploiting the power of meta‐analysis, we overcome many limitations of “omics” research in humans. Specifically, large sample sizes are required to detect changes with small effect sizes, which is the case of age (Su et al., 2015; Voisin et al., 2021) and exercise (Amar et al., 2021; Jacques et al., 2019; Pillon et al., 2020; Voisin et al., 2015)‐related changes in muscle OMIC profiles. All bioinformatics and statistical analyses were performed using the R statistical software.

### 4.1 Data mining

#### 4.1.1 Description of muscle DNA methylation and mRNA expression datasets

First, we gathered all existing DNAm and mRNA expression datasets from our laboratory and our collaborators’, in conjunction with public repositories, to assemble an exhaustive database of DNAm and mRNA expression profiles in muscle (Figure 1 and Tables S1 and S2). We focused exclusively on *microarray* experiments, as they are widely used, scalable (so individual datasets have larger sample sizes), and they measure the same CpGs or transcripts across datasets (so they are straightforward to meta‐analyze). We collected the methylomes of 1251 human samples from 16 datasets, profiled on the Illumina HumanMethylation platform (27 K, 450 K, and EPIC; Table S1 **)**, as well as the transcriptomes of 1926 samples from 21 datasets, profiled on Affimetrix, Illumina, and Agilent platforms (Table S2 **)**. For robustness, we only included datasets with >20 samples, with an age SD >5 years (age‐associated changes cannot be detected if age is invariant). Cohorts varied in their age range, health status, ethnicity, and potential treatments, so were adjusted for relevant covariates in the statistical analysis to detect age‐, CRF‐, exercise‐, and disuse‐related changes that are independent of undesirable confounders (see Tables S1 and S2 for the list of confounders adjusted in each cohort).

#### 4.1.2 Pre‐processing

We downloaded the raw IDAT files and pre‐processed all DNAm datasets, except for dataset GSE50498 for which we were missing batch information (we used the already pre‐processed matrix for this dataset). Details on the pre‐processing steps have been previously published (Voisin et al., 2021), and the preprocessing code is available on Sarah Voisin's Github account. First, we obtained β‐values defined as $\frac{\text{Methylated signal}}{\left(\text{Unmethylated signal}+\text{Methylated signal}+100\right)}$ . Then, we confirmed the sex of each sample by using the DNAm signal from the sex chromosomes (Aryee et al., 2014) and removed any sample whose annotated sex did not match the predicted sex (three samples removed across 16 datasets). We used the *ChAMP* pipeline (Tian et al., 2017) to preprocess each dataset; we ensured all samples had <10% of probes with detection *p*‐value > 0.01, and only excluded probes with missing β‐values, with a detection *p*‐value > 0.01, or with a bead count < 3 in more than 5% of samples. We removed non‐CG probes, SNP‐related probes (Zhou et al., 2017), and probes aligning to multiple locations; for datasets containing males and females, probes located on the sex chromosomes were also removed. Then, a β‐mixture quantile normalization method was applied to adjust for the Type I and Type II probe designs for methylation profiles generated from the HM450 and HMEPIC arrays. To identify technical and biological sources of variation in each individual dataset, singular value decomposition was performed. In all pre‐processed datasets, both the plate and the position on the plate were identified as significant technical effects. Thus, all β‐values were converted to M‐values, and the ComBat function from the *sva* package (Leek et al., 2012) was used to adjust directly for these technical artifacts.

We downloaded the already pre‐processed mRNA expression datasets, resolved any gene ID ambiguity (e.g., outdated gene names), and averaged the expression of transcripts annotated to the same EntrezID gene.

### 4.2 Identifying age‐related changes in the muscle methylome and transcriptome

#### 4.2.1 EWAS and TWAS meta‐analysis of age

To determine whether exercise training can slow down/reverse OMIC aging in human muscle, we first need to know which CpGs and mRNAs change during aging, in which direction, and to what extent. Therefore, we first conducted independent EWAS or TWAS of age in each methylation or transcription dataset. Details on the EWAS pipeline are available elsewhere (Voisin et al., 2021), and TWAS was conducted in a similar manner. The code for each EWAS and each TWAS is available on Sarah Voisin's Github account. Briefly, we regressed the DNAm level for each CpG (or the mRNA expression level for each transcript) against age, and adjusted the models for dataset‐specific covariates known to influence DNAm or mRNA expression levels (e.g., sex, ethnicity). We then conducted a random‐effects meta‐analysis to pool the summary statistics at each CpG and mRNA across datasets. Not all CpGs were present in all DNAm datasets, and not all mRNAs were present in all transcriptional datasets and restricted the analysis to the 595,541 CpG sites present in at least 10 of the 16 DNAm cohorts, and we restricted the analysis to the 16,657 genes present in at least 15 of the 21 datasets. Meta‐analysis was carried out using *metafor* (Viechtbauer, 2010) using “EB” (Empirical Bayes) as the residual heterogeneity estimator, 0.5 as the step length, 10,000 iterations, and an accuracy of 1^−8^ in the algorithm that estimates τ^2^. CpGs and mRNAs that showed a meta‐analysis false discovery rate (FDR) < 0.005 (Benjamin et al., 2017) were considered age‐related and selected for downstream analyses.

#### 4.2.2 Over‐representation analysis of ontologies (molecular pathways, human phenotypes)

To gain insights into the cellular and physiological consequences of aging on the muscle methylome and transcriptome, we tested whether genes belonging to canonical pathways (CP gene set in MSigDB), expression signatures of genetic and chemical perturbations (CGP gene set in MsigDB), gene ontology terms (GO gene set in MsigDB), and human phenotype ontologies (HPO gene set in MsigDB) were over‐represented among the age‐related CpGs and mRNAs. Over‐representation analysis (ORA) was performed with the *missmethyl* package (Phipson et al., 2016; Maksimovic et al., 2021) for DNAm, using all 595,541 tested CpGs as the background; ORA was performed with the *clusterProfiler* package (Wu et al., 2021) for transcription, using all 16,657 genes as the background. The ORA was restricted to gene sets containing 10–500 genes to limit type I error rate. Gene sets showing an FDR <0.005 (Benjamin et al., 2017) were considered significantly overrepresented.

#### 4.2.3 Confounding by changes in muscle cell type proportions

We used the same ORA technique to estimate whether age‐related changes in DNAm signal were potentially confounded by changes in muscle cell type proportions. We created a gene set containing markers genes for muscle cell types identified in a recent single‐cell transcriptional study of human muscle (Rubenstein et al., 2020; marker genes for muscle endothelial cells, smooth muscle cells, pericytes, FAP cells, PCV endothelial cells, satellite cells, FBN1 FAP cells, NK cells, myeloid cells, B cells, and T cells were from the “Rubenstein_skeletal_muscle” gene set in MSigDB, while marker genes for type I and type II fibers were downloaded directly from the original paper's supplementary table). Cell types showing an FDR <0.005 (Benjamin et al., 2017) were considered significantly overrepresented.

#### 4.2.4 Integration of aging methylome and transcriptome

We used the same ORA technique to estimate whether there was a significant overlap between age‐related changes at DNAm and mRNA expression levels. Age‐related differentially methylated genes (DMGs) were used as a gene set in the ORA for transcription, and age‐related differentially expressed genes (DEGs) were used as a gene set in the ORA for DNAm.

### 4.3 Estimating the effects of CRF, exercise training, and muscle disuse on the aging methylome and transcriptome

All analyses described henceforth have been conducted on the age‐associated Differentially Methylated Positions (DMPs) and Differentially Expressed genes (DEGs) identified in Step 1.

#### 4.3.1 Cardiorespiratory fitness

We focused on the muscle datasets for which information on baseline maximal oxygen uptake (VO_2max_) was available (Table 1). We only included datasets with baseline VO_2max_ SD >5 mL/min/kg (CRF‐associated changes cannot be detected if there is no variability in baseline CRF between participant). VO_2max_, measured during a graded exercise test, is considered the gold‐standard measurement of CRF.

We applied the same EWAS and TWAS meta‐analysis pipeline described previously but regressing DNAm or mRNA expression levels against VO_2max_. We then performed meta‐analysis for each aging CpG and transcript across datasets and adjusted for multiple testing. Aging CpGs or mRNAs that were associated with VO_2max_ at FDR <0.005 (Benjamin et al., 2017) were considered significant.

#### 4.3.2 Exercise training

For DNAm, we focused on the muscle datasets that came from exercise training studies (Table 2). We included all types of exercise training interventions (aerobic training, high‐intensity interval training, resistance training) as our aim was to test whether exercise in general could counteract the effect of age on aging OMIC profiles. We applied the same EWAS meta‐analysis pipeline described previously but looking at changes in DNAm levels after exercise training (i.e. regressing DNAm levels against Timepoint (PRE/POST training). Then, we performed the meta‐analysis for each aging CpG across datasets and adjusted for multiple testing. Aging CpGs whose DNAm levels changed following exercise training at FDR <0.005 (Benjamin et al., 2017) were considered significant.

For mRNA expression, we extracted summary statistics at age‐related mRNAs from a recently published meta‐analysis of exercise‐induced transcriptional changes (Amar et al., 2021). For each transcript, we used the summary statistics from the model selected by the authors (all data came from the meta_analysis_input.RData file uploaded by the authors on their Github page).

#### 4.3.3 Muscle disuse

There were no available muscle immobilization studies that profiled DNAm patterns in human muscle, so we could not estimate the effect of muscle disuse on age‐related DNAm patterns.

For mRNA expression, we extracted summary statistics at age‐related mRNAs from a published meta‐analysis of disuse‐induced transcriptional changes (Pillon et al., 2020). We used summary statistics sent by the authors upon correspondence with them.

### 4.4 Transcriptomic integration of age, CRF, exercise, and disuse

To contrast the effects of age, CRF, exercise training, and muscle disuse, we used multi‐contrast enrichment analysis as implemented in the *mitch* package (Kaspi & Ziemann, 2020). As recommended in the package, we first created a score to represent the importance of the differential gene expression for each transcript and each contrast:

$$\text{Score}=\mathrm{sign}\left(\log_{2}\mathrm{FC}\right)\times {-\log}_{10}\left(\text{pvalue}\right)$$

For example, the *FEZ2* gene decreased in expression with age at a magnitude of log_2_FC = −0.12 per year of age at a *p*‐value of $1.24\times 10^{-10}$. The age score for *FEZ2* was therefore $-1\times {-\log}_{10}\left(1.24\times 10^{-10}\right)=-9.9.$

We then performed multi‐contrast enrichment analysis using the same gene sets as in the ORA analysis described above (canonical pathways, expression signatures of genetic and chemical, gene ontology terms, and human phenotype ontologies), using the default parameters in the mitch_calc() function. Gene sets showing an FDR <0.005 were considered significant.

### 4.5 Visualization tools

Pairwise correlation plots using Spearman correlations were graphed using the *GGally* package, heatmaps were graphed using the *pheatmap* package after scaling the effect sizes for age, VO_2max_, exercise and/or disuse, and forest plots were graphed using the *metafor* package.

## AUTHOR CONTRIBUTIONS

S.V. performed the bioinformatics and statistical analyses, prepared figures, and wrote the manuscript, with contributions from K.S.; S.L., M.J., N.R.H., K.J.A., L.M.H., L.R.G., V.G.C., T.D., and J.M.T. collected samples and provided data for the Gene SMART study; M.E.L. provided data for the EpiTrain study; C.W., G.D., R.I., and C.M. provided data for the EXACT study; O.H. and O.A. provided data for the Malmö Prevention and MSAT studies; A.E.H., P.P., K.P., and M.O. provided data for the FTC study; S.B. and M.T. provided data for the EPIK study; C.K.D. provided data for the E‐MTAB‐11282 study; A.P.S. provided data for the GSE114763 and CAUSE studies; N.E. supervised the work and provided detailed feedback on the analyses and the manuscript. All authors contributed to editing and finalization of the manuscript.

## CONFLICT OF INTEREST STATEMENT

The authors declare that they have no conflict of interest.

## Supporting information


FigureS1

TableS1
