# Supplementary material for: Exercise is associated with younger methylome and transcriptome profiles in human skeletal muscle
Source: Aging Cell. 2023 May 2;23(1):e13859. doi: 10.1111/acel.13859 (PMC10776126; doi:10.1111/acel.13859)
Supplement: Supplementary file 1 — FigureS1 [file ACEL-23-e13859-s002.docx]

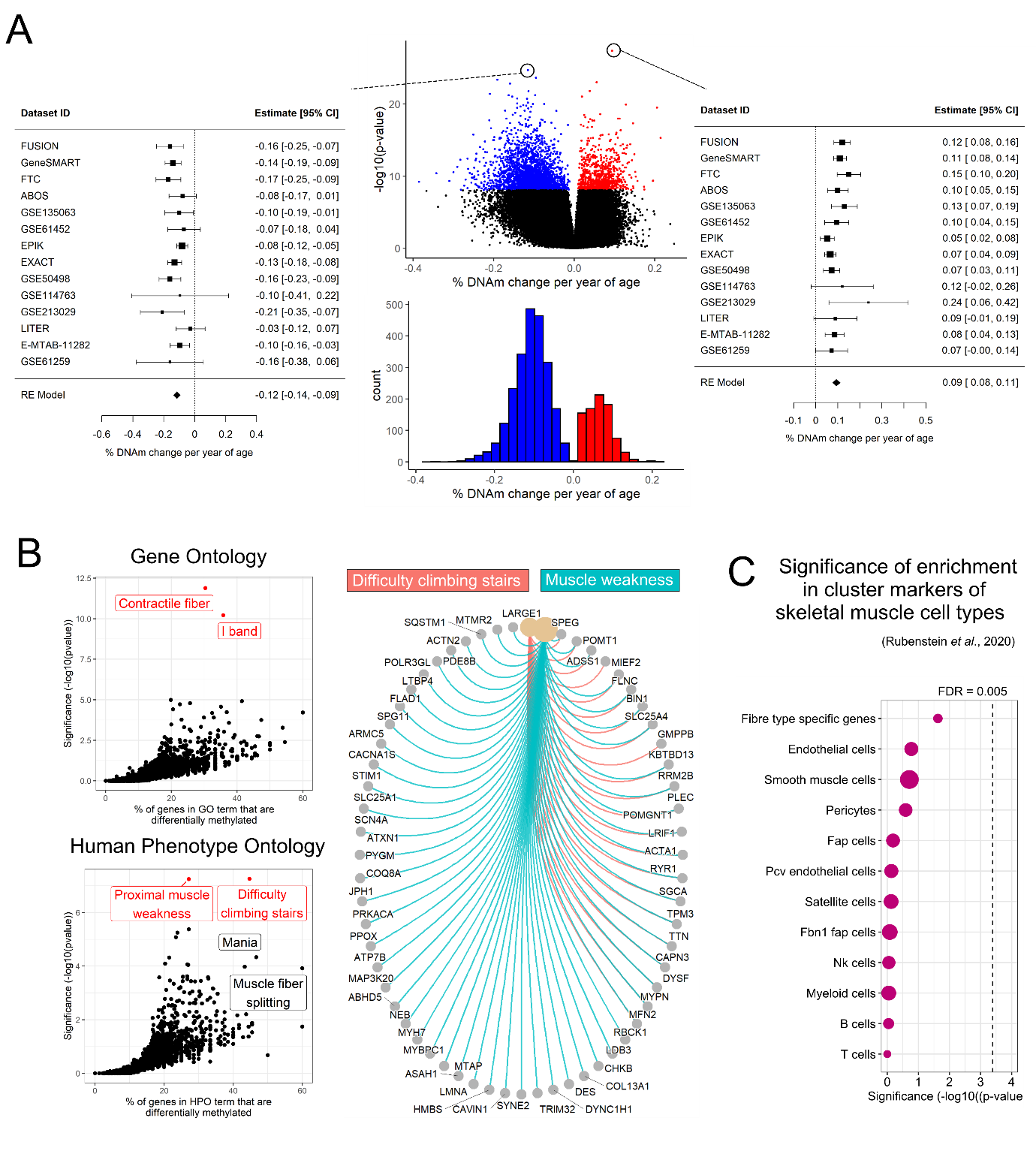


**Supplementary Figure 1**. **EWAS meta-analysis of age reveals small, widespread DNA methylation changes at genes encoding structural components of muscle**. A) Volcano plot of the effect size (% DNAm change per year of age) and significance of age for each CpG included in the meta-analysis (pooled results of 16 independent muscle datasets); colored dots are CpGs that are differentially methylated at a false discovery rate (FDR) < 0.005; we highlighted the top hypo- (left) and hyper- (right) methylated CpG in forest plots to show the estimates and confidence intervals in each dataset. B) Overrepresentation analysis of the age-related differentially methylated positions for Gene Ontology, and Human Phenotype Ontology (HPO) from MSigDB; pathways in red are significant at an FDR < 0.005. The circular plot displays genes from the two significant HPOs (“difficulty climbing stairs”, and “muscle weakness”) that showed age-related alterations at the DNAm level. C) Dotplot of the enrichment of DMPs in marker genes for 12 cell types identified in a single-cell sequencing study of human skeletal muscle^38^.


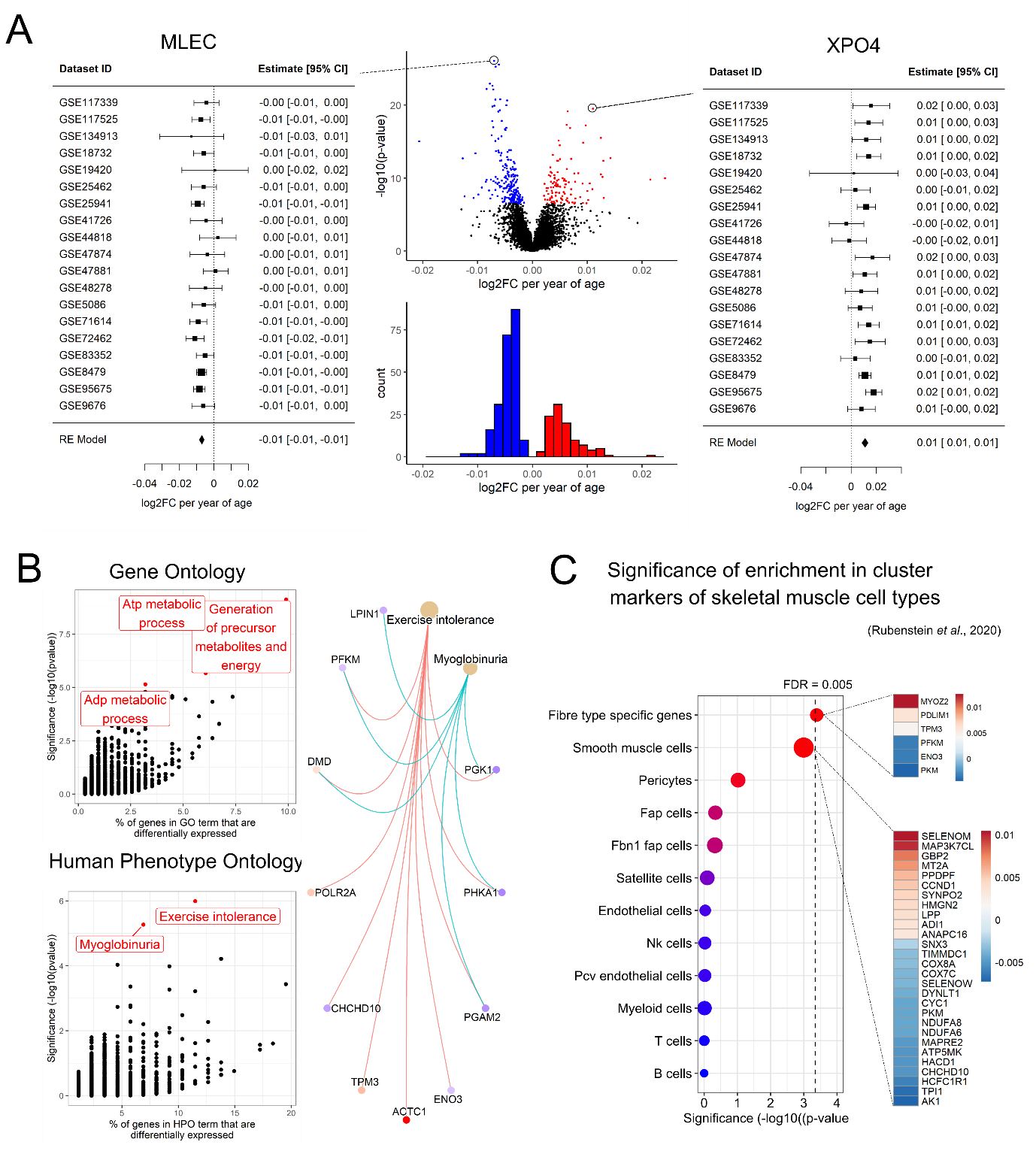


**Supplementary Figure 2**. **TWAS meta-analysis of age reveals mRNA expression changes at genes involved in mitochondrial function and energy production**. A) Volcano plot of the magnitude (log2 fold-change (log2FC) per year of age) and significance of age effects for each gene included in the meta-analysis (pooled results of 21 independent muscle transcriptomic datasets); coloured dots are genes that are differentially expressed at a false discovery rate (FDR) < 0.005; we highlighted the top down- (left) and up- (right) regulated genes in forest plots to show the estimates and confidence intervals in each dataset. B) Overrepresentation analysis of the age-related differentially expressed genes (DEGs) for Gene Ontology (GO), and Human Phenotype Ontology (HPO) from MSigDB; pathways in red are significant at an FDR < 0.005. The circular plot displays DEGs from the two significant HPOs (“exercise intolerance”, and “myoglobinuria”). C) Dotplot of the enrichment of DEGs in marker genes for 11 cell types identified in a single-cell sequencing study of human skeletal muscle^38^. The heatmap displays the log2FC of the DEGs that are marker genes for smooth muscle cells.


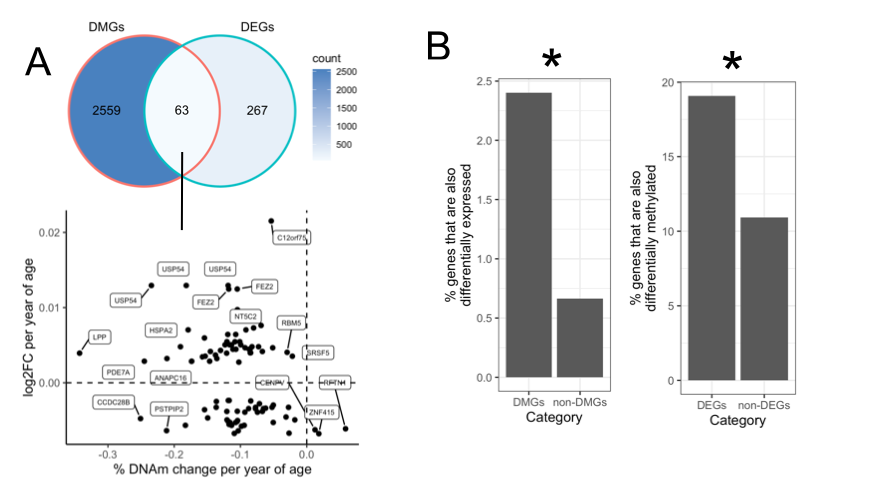


**Supplementary Figure 3**. **Overlap between age-related differentially methylated genes (DMGs) and differentially expressed genes (DEGs)**. A) The 3,168 DMPs were located in 2,622 unique genes, 63 of which were also DEGs. On the bottom graph, each dot represents a DMP-DEG pair, with the change in DNAm level (x-axis) and mRNA level (y-axis) per year of age. Note that some genes appear multiple times on the plot as multiple DMPs could be annotated to the same gene (e.g. FEZ2, USP54). B) Left, proportion of genes within DEGs and non-DEGs that are also differentially methylated with age; right, proportion of genes within DMGs and non-DMGs that are also differentially expressed with age. *p < 0.005 in over-representation test (Fisher’s exact test).


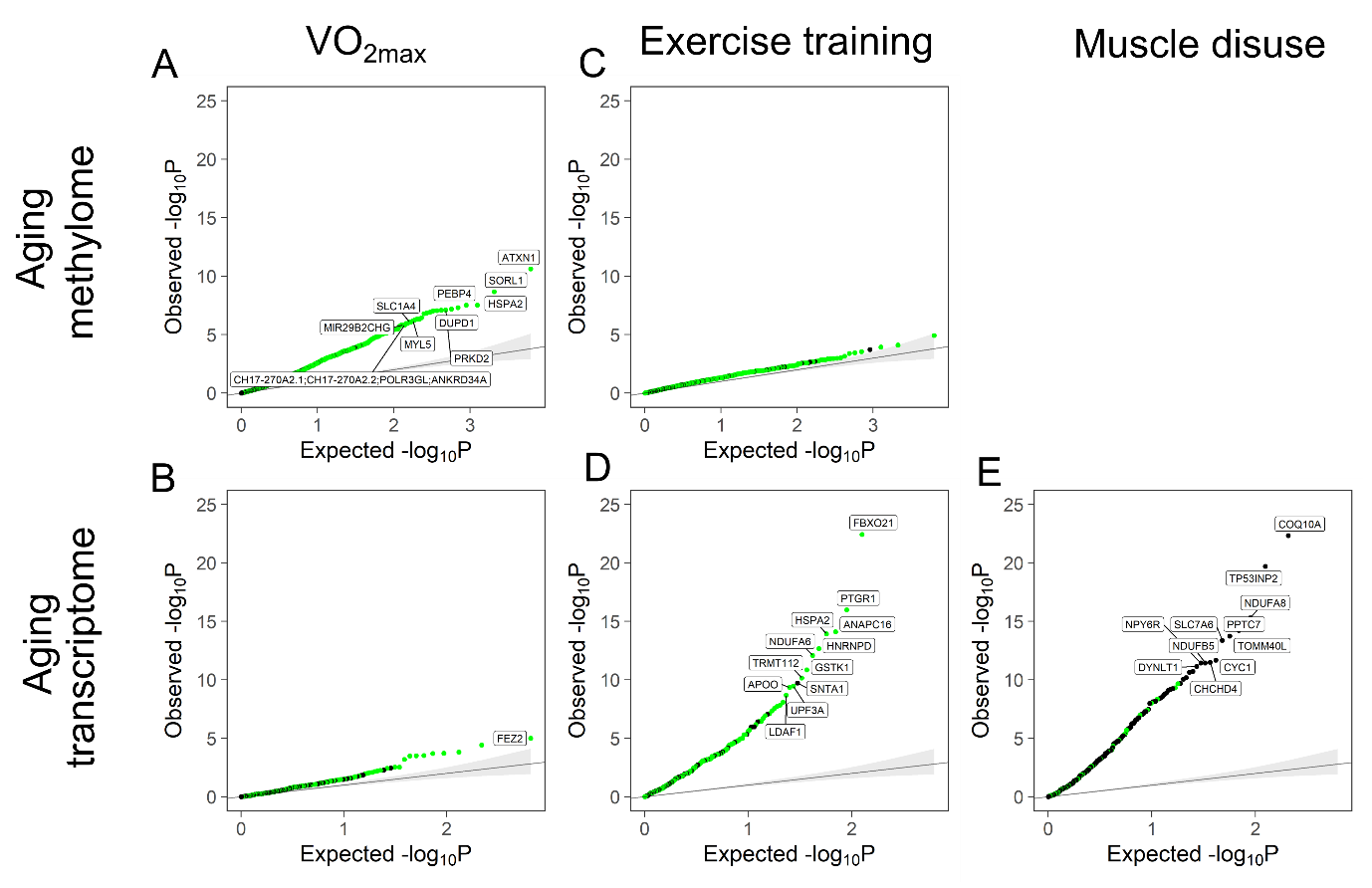


**Supplementary Figure 4**. **Quantile-quantile plot of p-values for the effect of VO_2max_, exercise training and muscle disuse on the aging methylome and transcriptome**. The observed p-values for each age-related DMP or DMG are sorted from largest to smallest and plotted against expected values from a theoretical χ2-distribution. If some observed p-values are clearly more significant than expected under the null hypothesis, points will move towards the y-axis; green points represent DMPs or DEGs for which the effect of age contrasts with the effect of VO_2max_ or exercise or disuse, while black points represent DMPs or DEGs for which the effect of age is in line with the effect of VO_2max_ or exercise or disuse.
